# Titanium-interlayer mediated hydroxyapatite coating on polyetheretherketone: a prospective study in patients with single-level cervical degenerative disc disease

**DOI:** 10.1186/s12967-020-02688-z

**Published:** 2021-01-06

**Authors:** Ce Zhu, Miaomiao He, Lili Mao, Tao Li, Li Zhang, Limin Liu, Ganjun Feng, Yueming Song

**Affiliations:** 1 Department of Orthopedics Surgery and Orthopedics Research Institute, West China Hospital, Sichuan University, No. 37 Guoxue Road, Chengdu, 610041 Sichuan China; 2 Department of Spine Surgery, The Affiliated Hospital of Southwest Medical University, Luzhou, China; 3 Analytical & Testing Center, Sichuan University, Chengdu, China; 4 Department of Ultrasound, Hospital of Traditional Chinese Medicine Affiliated to Southwest Medical University, Luzhou, China

**Keywords:** Polyetheretherketone, Titanium, Hydroxyapatite, Cage, Cervical degenerative disc disease

## Abstract

**Background:**

Currently, there are limited reports regarding investigation of the biological properties of polyetheretherketone (PEEK) coated with titanium (Ti) and hydroxyapatite (HA) in human. The objective of this study is to evaluate the in vivo response of the PEEK cages coated with Ti and HA versus uncoated PEEK cages after anterior cervical discectomy and fusion (ACDF) in patients with single-level cervical degenerative disc disease (CDDD).

**Methods:**

Twenty-four patients with PEEK cages coated with Ti and HA (PEEK/Ti/HA group) were matched one-to-one with patients with uncoated PEEK cages (PEEK group) based on age, gender, and operative segment. All patients had been followed up for more than 2 years. Radiological assessments included intervertebral height (IH), C2-7 angle (C2-7a), segmental alignment (SA), and fusion rate. Clinical parameters included Visual Analogue Scale (VAS) and Japanese Orthopedic Association (JOA) scores.

**Results:**

There was no statistical difference in SA, IH, and C2-7a between the two groups before and after surgery and all these parameters were restored postoperatively. The fusion rate of PEEK/Ti/HA group was significantly higher than PEEK group at 3-month post-operation (87.5% vs. 62.5%). At the last follow-up, the fusion rate of the both groups achieved 100%. The VAS and JOA scores were comparable between two groups and improved postoperatively.

**Conclusions:**

In patients with single-level ACDF, PEEK cage coated with Ti and HA provided a higher fusion rate than uncoated PEEK cage at 3-month post-operation, while both two cages could achieve solid osseous fusion at the last follow up. Compared with the uncoated PEEK cage, PEEK/Ti/HA cage yielded similar favorable segmental and overall cervical lordosis, IH, and clinical outcomes after the surgery.

## Background

Anterior cervical discectomy and fusion (ACDF) was first described by Robinson and Smith and popularized by Cloward in the 1950s [1, 2]. It is the most widely used method for the surgical treatment of cervical degenerative disc disease (CDDD) via its positive fusion rate and patient self-assessment outcomes [3, 4]. The implant for replacement of diseased disc can provide a mechanical support between the two endplates as well as facilitate bone growth between the two vertebral bodies [5]. Autograft is considered to be the gold standard for ACDF because of its favorable biocompatibility and high fusion rates without immunogenicity. However, it needs a second surgical site which increases the operative time and blood loss as well as the potential risks for the donor site such as pain, hematomas, seromas, infections and fractures [6].

The complications of autograft aforementioned aroused the exploration of new bone substitutes that could provide sufficient mechanical and biologic properties. The two main materials currently used are titanium (Ti) alloys and polyetheretherketone (PEEK). Titanium alloys are advantageous in their excellent corrosion resistance, high mechanical strength and cytocompatibility, but they are susceptible to stress shielding and may result in subsidence due to its high elastic modulus [5]. In addition, the inherent high radiopacity of titanium alloys may produce metal artifacts in computed tomography (CT) images, which would interfere the assessment of fusion results.

PEEK is a semi-crystalline, synthetic thermoplastic polymer that exhibits excellent fracture toughness, thermal stability and environmental resistance [7]. PEEK has an elastic modulus similar to that of natural bone, which prevent the stress shielding that is often observed in titanium alloys implants. Furthermore, the radiolucency of PEEK helps surgeons observe the bone healing around the implants. Nonetheless, the osteoconductive and osteoinductive properties of the PEEK are relatively unsatisfactory due to its bioinert surfaces. To improve osteoblast responses and bone integration of the PEEK, surface modifications of the PEEK have been proven to be an effective strategy [8].

Hydroxyapatite (HA) is a bioactive calcium phosphate with similarities to the mineral phase of natural bone, which has been used for the coating on PEEK to improve its osseointegration with promising results [9, 10]. The coating techniques include plasma spraying, spin coating, sandblasting, diazonium chemistry, sputtering and etc. [8, 10, 11]. Among them, plasma spraying is the most widely used commercial coating technique with good reproducibility and high deposition rates [7, 12]. Nevertheless, the adhesion strength of plasma-sprayed HA coatings on PEEK is very poor [10]. Stübinger et al. [13] coated HA films on the PEEK by adding a Ti intermediate layer between the HA film and PEEK using a plasma spraying technique, which demonstrated that the plasma-sprayed Ti and HA coatings on PEEK displayed a significant improvement of osseointegration in sheep.

However, to our knowledge, the studies concerning the investigation of the biological properties of PEEK coated with Ti and HA in human are limited. In the present study, a PEEK cage with Ti and HA coatings using a plasma spraying technique was prepared and applied to ACDF for the treatment of patients with single-level CDDD. The objective of this study is to evaluate the in vivo response of the PEEK cages coated with Ti and HA versus those uncoated PEEK cages after ACDF in patients with single-level CDDD.

## Methods

### Materials and characterizations

**Fig. 1:**
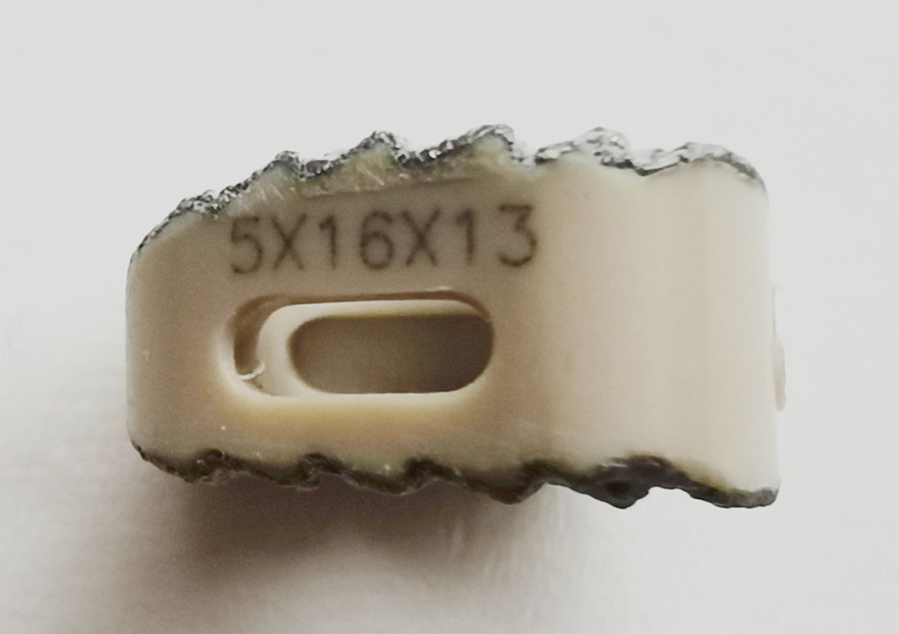
PEEK cage coated with Ti and HA

The PEEK cages coated with Ti and HA respectively by plasma spray were supplied by WEGO Holding Co., Ltd. China (Fig. 1). The top and cross-section view were observed by scanning electron microscope (SEM, JSM-7500F, JEOL, Japan), and the corresponding element was analyzed by energy disperse spectroscopy (EDS, JSM-7500F, JEOL, Japan) coupled with SEM. X-ray diffraction (XRD, EMPYREAN, PANalytical B.V., Holland) was carried out to confirm the surface phases on PEEK cage. Compressive testing was performed using universal mechanical testing machine (MTS, model E45, America) with a loading speed of 4 mm/min.

### Clinical assessments

This was a prospective and non-randomized study. This study was approved by the ethics committee of West China Hospital of Sichuan University and informed consent was obtained from the patients. The patients gave written consent for publication of their clinical details and clinical images. Twenty-four patients with single-level CDDD between August 2016 and October 2017 in our department who underwent ACDF with PEEK cages coated with Ti and HA (PEEK/Ti/HA group) were included in the study. For comparison, 24 patients who underwent single-level ACDF with uncoated PEEK cages (PEEK group) were matched one-to-one to the patients in the PEEK/Ti/HA group. Matching characteristics included age (within 1 year of one another), gender, and operative segment.

The inclusion criteria were: (1) age ≥ 18 years, (2) radiculopathy and/or myelopathy from single-level cervical disc herniation, (3) no response to 3 months of non-surgical management, and (4) no previous spine surgery. Exclusion criteria included previous spine surgery, active infection, and inflammatory spondyloarthropathies.

All operations were performed by the same surgeon under general anesthesia. All procedures were performed through a transverse skin incision on the right side of the neck. Discectomy was performed using a standard anterior cervical approach [1, 14]. The vertebral body cartilage endplates at the treated level were resected by high-speed burr and curette. The osteophyte located at the posterior edge of the vertebral body, and the ruptured posterior longitudinal ligament were completely removed. The cage with an appropriate size filled with morselized bone from the local decompression was carefully implanted at the decompressed intervertebral space. An ATLANTIS Anterior Cervical Plate System (Medtronic Sofamor Danek USA, Inc. Memphis, TN) was used to achieve immediate stabilization. After surgery, patients were braced in a cervical collar for about 6 weeks.

**Fig. 2:**
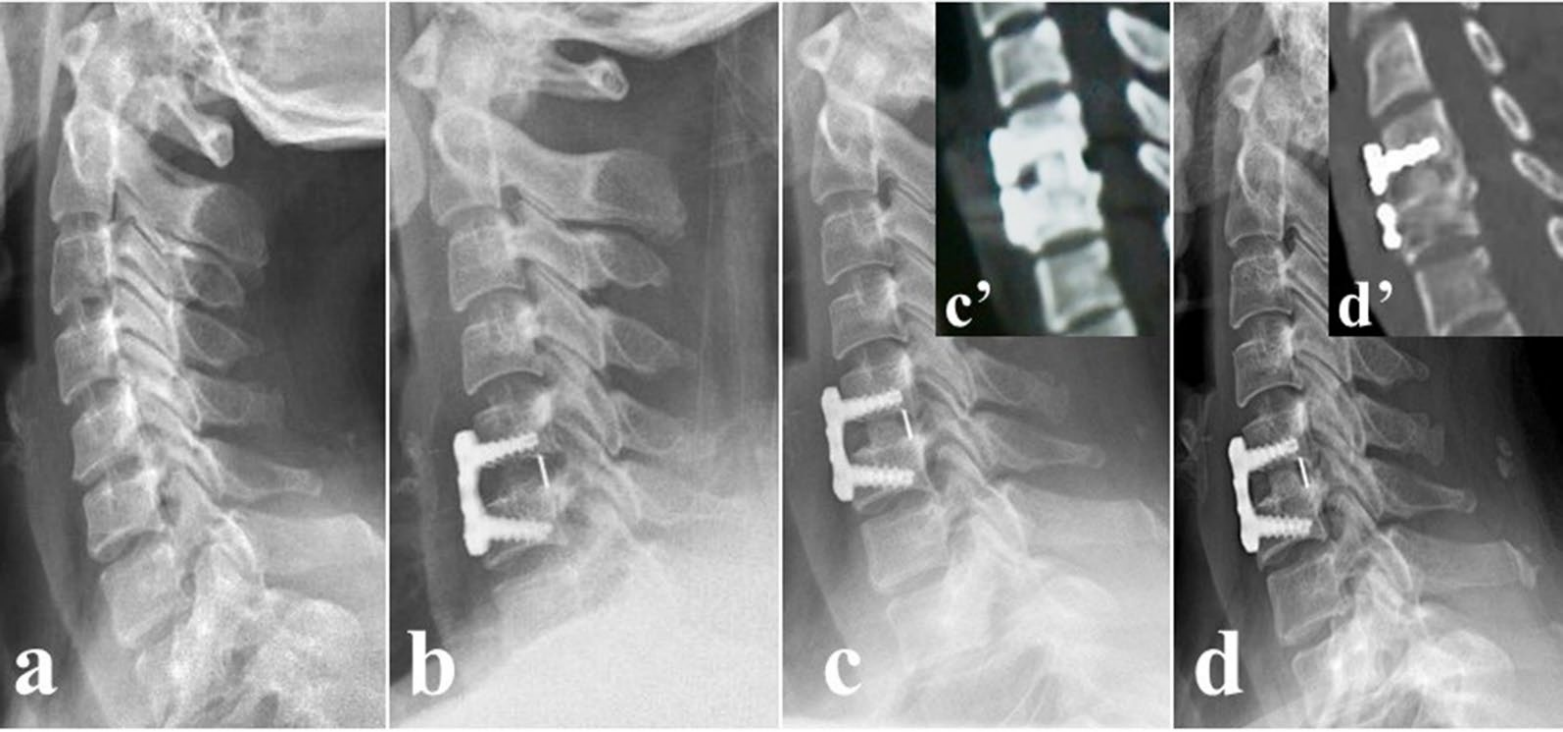
A 46-year-old woman undergoing anterior cervical discectomy and fusion surgery with a PEEK/Ti/HA cage. **a** Preoperative plain lateral radiographs image. **b** Postoperative lateral radiograph image. **c** Lateral radiograph image at 3-month follow-up. **c’** CT scans at 3-month follow-up. **d** Lateral radiograph image at the final follow-up. **d’** CT scans at the final follow-up. PEEK, polyetheretherketone; Ti, titanium; HA, hydroxyapatite; CT, computerized tomography

**Fig. 3:**
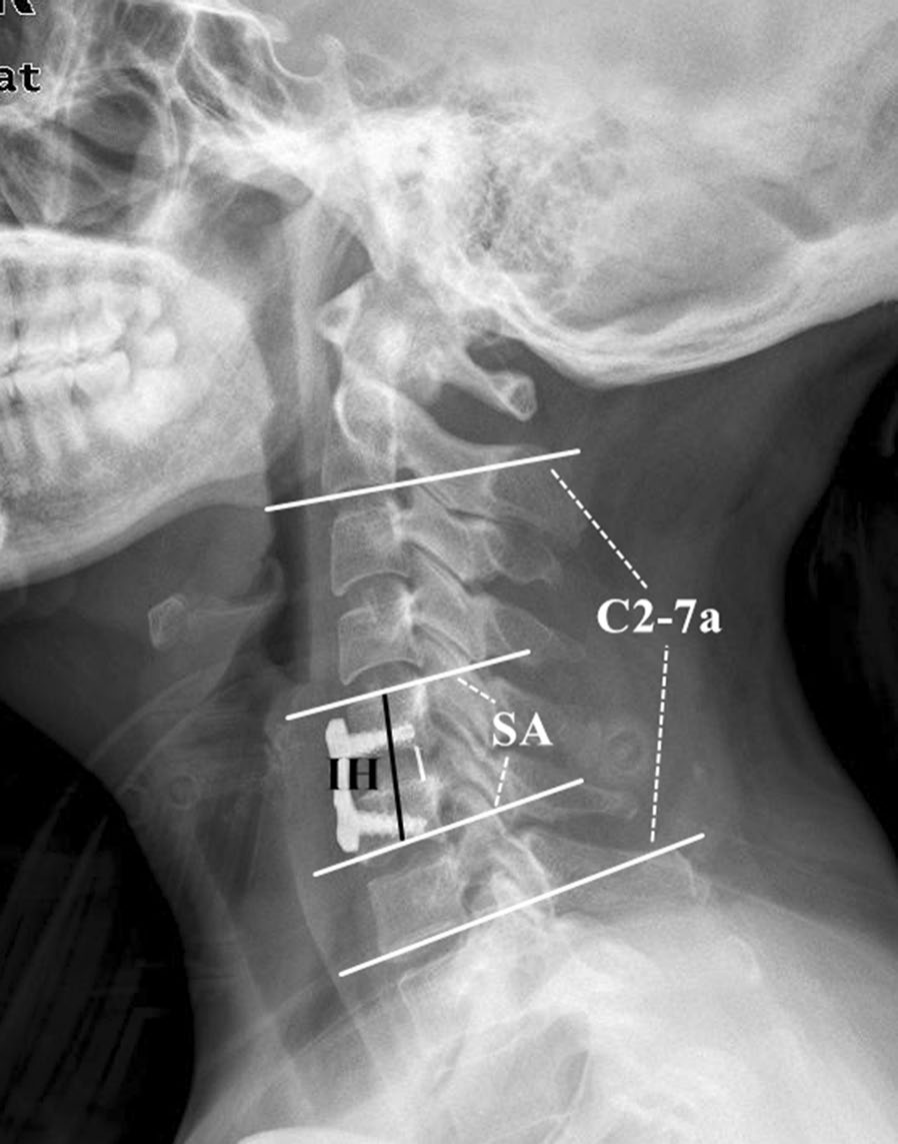
Representation of radiographic measurements: IH (intervertebral height), the distance from the midpoint of the upper endplate of the upper vertebral body to the midpoint of the lower endplate of the lower vertebral body; C2-7a (C2-7 angle), the Cobb angle between the lower endplate of C2 and C7; SA (segmental alignment), the Cobb angle between the superior endplate of the upper vertebra and the inferior endplate of the lower vertebra of the implanted level

Frontal and lateral radiographs and three-dimensional CT scans (3d-CT) of the cervical spine were obtained at baseline and the 3-month and final follow-up after surgery (Fig. 2). The following parameters were observed on lateral neutral radiographs (Fig. 3): intervertebral height (IH), the distance from the midpoint of the upper endplate of the upper vertebral body to the midpoint of the lower endplate of the lower vertebral body; C2-7 angle (C2-7a), the Cobb angle between the lower endplate of C2 and C7; segmental alignment (SA), the Cobb angle between the superior endplate of the upper vertebra and the inferior endplate of the lower vertebra of the implanted level. Negative values indicated kyphosis while positive values indicated lordosis. Subsidence was defined as loss of height of more than 3 mm [15]. The fusion status was evaluated on 3d-CT by the 5‑grade criteria proposed by Brantigan et al. [16]. The Grades 4 or 5 were defined as fused while Grade 1 or 2 as unfused and Grade 3 was uncertain. All radiological parameters were measured using picture archiving and communication systems (PACS) by 2 attending spinal surgeons who were not involved in the surgery, and the average value of their measurements was used for analysis.

The Japanese Orthopedic Association (JOA) scores and Visual Analogue Scale (VAS) were used for the evaluation of clinical outcomes before surgery, 3 months after surgery and at the final follow-up.

All data were analyzed by using SPSS software (version 22.0; IBM Corp., Armonk, NY, USA). All values are presented as the mean ± standard deviation. Quantitative data were analyzed by using Student’s t-test or the Mann–Whitney U test as appropriate. Categorical data were analyzed by using the χ^2^ test or Fisher’s exact test. Statistical significance was set at P < 0.05.

## Results

**Fig. 4:**
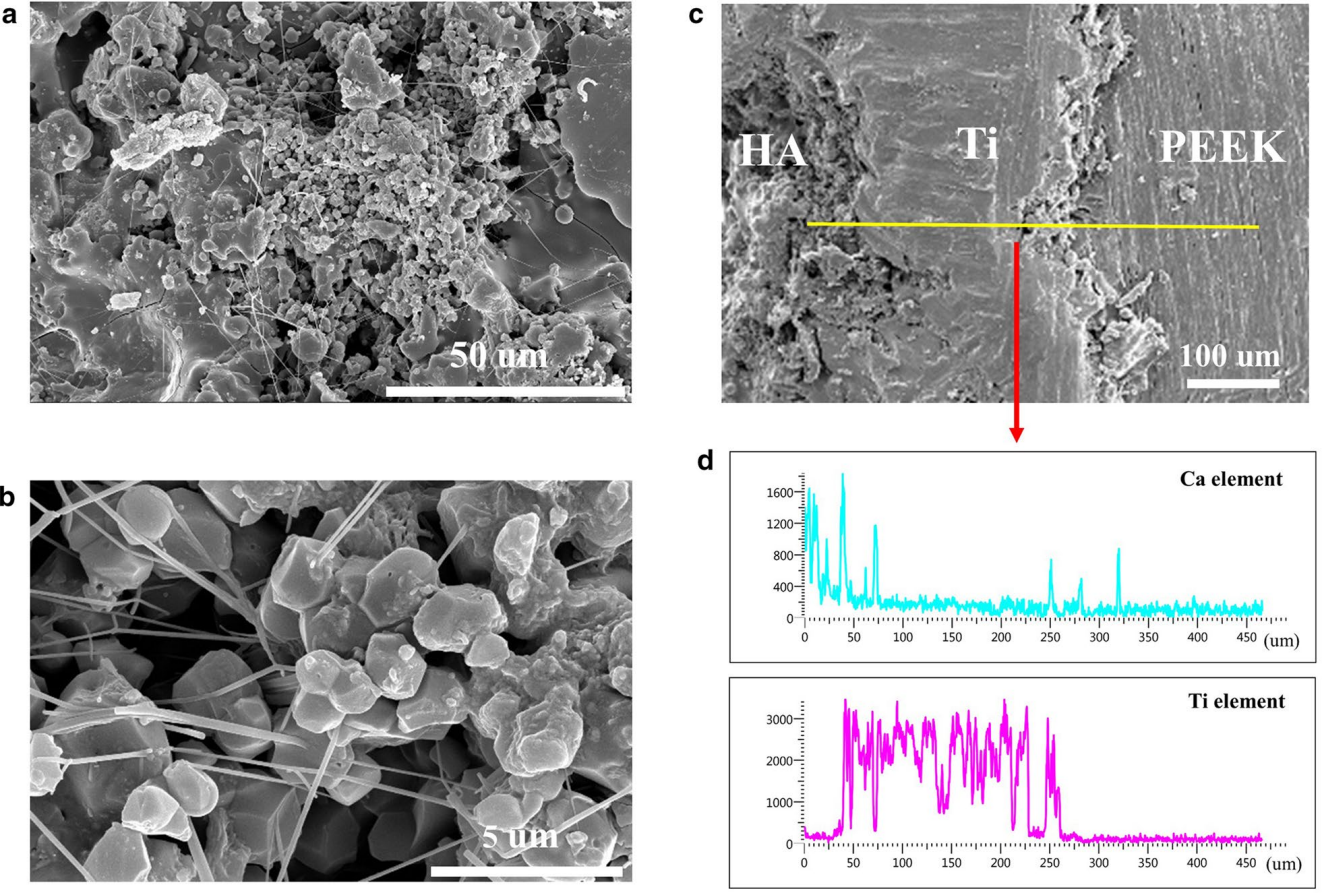
SEM images of the samples surface with low (**a**) and high (**b**) magnification; **c** the cross-section SEM images of the samples; **d** the corresponding EDS line scanning

Figure 4a, b showed the SEM images with different magnifications of the PEEK cage surface. As shown in the images, HA particles with micron sizes stacked on the surface of samples. The SEM image in the cross-section of the cage (Fig. 4c) indicated that Ti layer of approximately 200 µm in thickness was located between the PEEK matrix and HA coating, in accordance with the coating treatment sequence. The corresponding EDS line scan (Fig. 4d) in the cross section showed that from the top surface to the matrix, the compositions showed up in the order of HA, HA + Ti, Ti, and PEEK matrix.

**Fig. 5:**
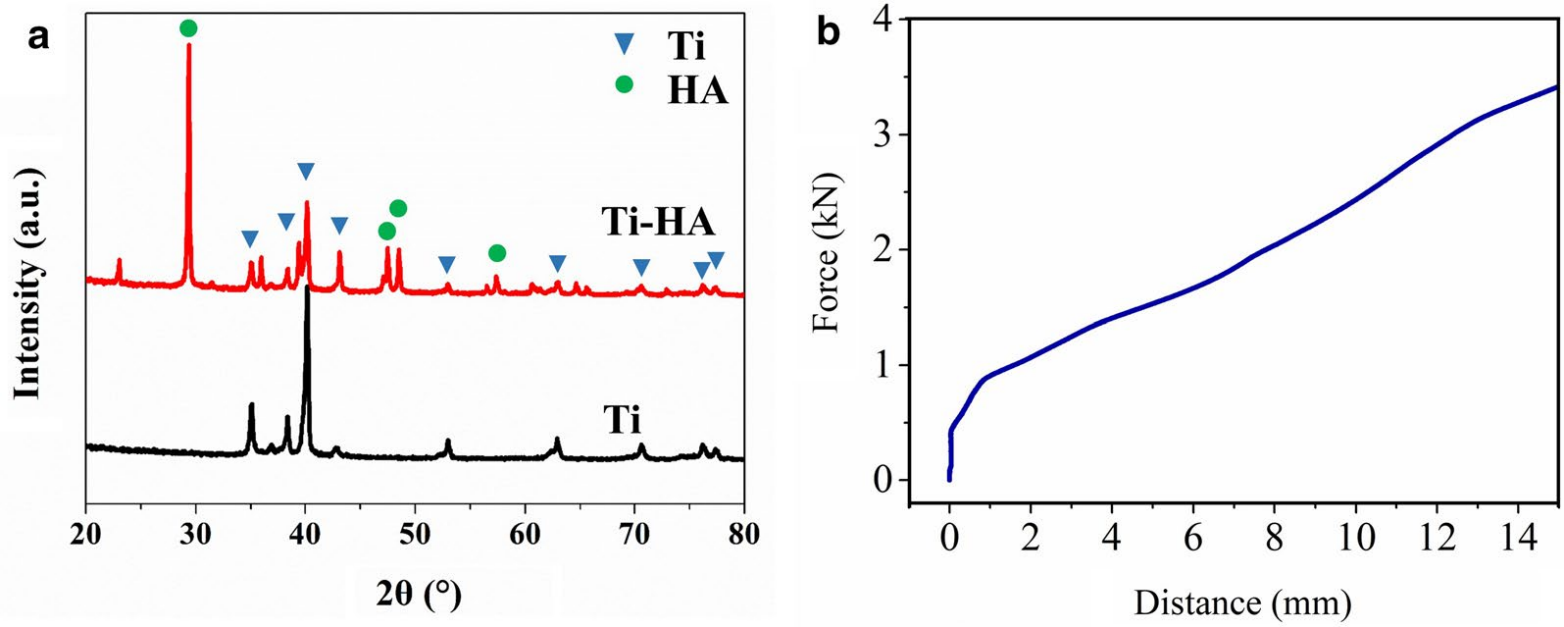
**a** XRD of the powder scrapped from coating on the surface of materials respectively; **b** the compressive force-distance curve of PEEK cage

The phase structure and crystallinity of the PEEK cage was investigated by XRD and compared with pure Ti (Fig. 5a). The diffraction peaks at 28.9° (210), 48.6° (320), and 48.01° (312) were detected which indicated the presence of HA (PDF# 09-0432). The characteristic peak of Ti (PDF# 44-1294) was also confirmed, indicating the coating was composited by Ti and HA. The compressive test of PEEK cage (Fig. 5b) showed the cage can bear a load of 0.43 kN, which can support the mechanical property required for the spine. The force over 0.43 kN would deform PEEK cage.

The average postoperative follow-up period time ranged from 24 to 41 months (mean 31.5 ± 6.2 months). Twenty-two patients (45.8%) had radiculopathy (PEEK: PEEK/Ti/HA = 12:10), 16 patients (33.3%) had myelopathy (PEEK: PEEK/Ti/HA = 7:9), and 10 patients (20.8%) had both radiculopathy and myelopathy (PEEK: PEEK/Ti/HA = 5:5). There was no significant difference between the two groups regarding their diagnoses (p = 0.806). The operative segments included C3/4, C4/5, C5/6 and C6/7 (4, 10, 22, 12, respectively). There were no significant differences in age, gender, smoker, operative time, and blood loss between the PEEK/Ti/HA group and PEEK group (Table 1).

**Table 1:** Patient demographic data

	PEEK/Ti/HA group	PEEK group	P
Age (year)	49.2 ± 5.7	49.0 ± 5.8	0.901
Gender (male/female)	14/10	14/10	1.000
Smoker	7/24	5/24	0.740
Operative time (min)	107.2 ± 15.6	108.5 ± 15.5	0.782
Blood loss (mL)	76.8 ± 18.0	74.2 ± 19.7	0.633

*PEEK* polyetheretherketone, *Ti* titanium, *HA* hydroxyapatite

* p < 0.05

The radiographic parameters in these two groups were listed in Table 2. There was no statistical difference in SA, IH, and C2-7a at baseline, 3-month and at the final follow-up after surgery between the two groups (P > 0.05). The SA and C2-7a improved significantly in both groups 3 months after surgery, and the effect remained at the final follow-up. The IH increased significantly in both groups postoperatively, but decreased at the final follow-up. The cage subsidence rate was the same in both two groups (1/24, 4.2%). The fusion rate of the patients in the PEEK/Ti/HA group was significantly higher than that of patients in the PEEK group at 3-month post-operation (87.5% vs. 62.5%, P < 0.05). However, the difference of fusion rate between the two groups at 6-month post-operation, 1-year post-operation and the last follow-up were not statistically significant (PEEK: PEEK/Ti/HA = 83.3%: 95.8%, 100%: 100%, 100%: 100%; P = 0.156, 1.000, 1.000; respectively).

**Table 2:** Radiological outcomes of the patients

	PEEK/Ti/HA group	PEEK group	P
SA (°)			
Pre-op	2.0 ± 1.1	1.8 ± 1.1	0.531
3 m post-op	6.2 ± 3.3*	6.0 ± 3.7*	0.841
Final follow-up	4.9 ± 2.2*	4.7 ± 2.5*	0.806
IH (mm)			
Pre-op	34.5 ± 1.5	34.7 ± 1.6	0.646
3 m post-op	36.7 ± 1.1*	37.1 ± 1.2*	0.215
Final follow-up	35.5 ± 1.1*^,#^	36.0 ± 1.1*^,#^	0.135
C2-7a (°)			
Pre-op	12.7 ± 7.4	13.0 ± 6.9	0.894
3 m post-op	18.0 ± 7.8*	19.3 ± 6.9*	0.533
Final follow-up	18.5 ± 8.3*	18.3 ± 10.0*	0.960

*PEEK* polyetheretherketone, *Ti* titanium, *HA* hydroxyapatite, *SA* sagittal alignment, *IH* intervertebral height, *C2-7a* C2-7 angle, *Pre-op* preoperative, *3 m Post-op* 3-month postoperative

* p < 0.05 compared with pre-op

^#^ p < 0.05 compared with 3 m post-op

No intergroup significant difference was found in terms of the clinical outcomes (Table 3). The JOA score and VAS score of the patients in both two groups were improved after the surgery.

**Table 3:** Clinical outcomes of the patients

	PEEK/Ti/HA group	PEEK group	P
JOA score			
Pre-op	10.5 ± 1.2	10.1 ± 1.1	0.217
3 m post-op	15.4 ± 0.8*	15.0 ± 0.9*	0.114
Final follow-up	15.8 ± 0.9*^,#^	15.5 ± 1.1*^,#^	0.407
VAS score			
Pre-op	7.9 ± 1.3	7.7 ± 1.5	0.772
3 m Post-op	2.5 ± 1.0*	2.3 ± 1.0*	0.690
Final follow-up	2.4 ± 1.1*	2.3 ± 1.1*	0.797

*PEEK* polyetheretherketone, *Ti* titanium, *HA* hydroxyapatite, *JOA* Japanese Orthopedic Association, *VAS* visual analogue scale, *Pre-op* preoperative, *3 m Post-op* 3-month postoperative

* p < 0.05 compared with pre-op

^#^ p < 0.05 compared with 3 m post-op

## Discussion

Previous studies demonstrated the benefits of Ti and/or HA on PEEK surfaces. Lu et al. [17] found the enhancement of adhesion, proliferation, and osteo-differentiation of rat bone mesenchymal stem cells (BMSCs) by introducing titanium ions into carbon-fiber-reinforced PEEK (CFR-PEEK) surface via plasma immersion ion implantation. Walsh et al. [18] reported that the titanium coating dramatically improved the shear strength at the bone-implant interface at 4 weeks and continued to improve with time compared with PEEK. Lee et al. [19] observed higher alkaline phosphatase (ALP) activity, calcium production, and bone sialoprotein (BSP) production of human bone marrow mesenchymal stem cells on the HA-coated PEEK implants than the bare PEEK group in vitro test. They also demonstrated the better biocompatibility and osseointegration of the HA-coated PEEK in vivo minipig model. Stübinger et al. [13] prepared a double-coated CFR-PEEK which made of a first Ti bond layer and a second hydroxyapatite top layer by air plasma spray. Compared to the uncoated PEEK/CFR-PEEK, Ti coating and HA coating, the double Ti–HA coating achieved the most favorable biomechanical and biological results in a sheep pelvic model.

So far, to the best of our knowledge, there is a limited published data related to osseointegration and biocompatibility of PEEK coated with titanium and HA in human. In this study, we successfully coated PEEK surfaces with rough titanium and hydroxyapatite layers together. And we sought to evaluate the clinical and radiographic outcomes of ACDF using PEEK cages coated with titanium and HA compared to a matched uncoated PEEK cages cohort with a minimal follow-up time of 2 years.

It is generally believed that fusion rate is a critical prognostic factor in ACDF. In the present study, patients in the PEEK/Ti/HA group achieved higher fusion rate than patients in the PEEK group (87.5% vs. 62.5%, P < 0.05) 3 months postoperatively. Meanwhile, solid osseous fusion was found in all the patients of the both two groups since 1-year post-operation (100% fusion rate). These results indicated an excellent fusion capability of the PEEK cages coated with Ti and HA. The reasons are both of biological and physical nature of the Ti and HA layers: first of all, the biocompatibility and osteoconductivity of Ti and HA is higher than that of PEEK, which have been proved by previous studies [13, 17–20]. In addition, the rough coating surface provides high initial fixation of the intervertebral space by increasing frictional forces and limiting micromotion [13, 21].

Cage subsidence is a common complication of ACDF which relates to kyphotic deformity, instrument failure and postoperative neurologic deterioration [22]. In our study, the cage subsidence rate was the same in both two groups (1/24, 4.2%). The IH in the PEEK/Ti/HA group increased from 34.5 mm preoperatively to 36.7 mm postoperatively, but decreased to 35.5 mm at final follow-up, while the IH in the PEEK group increased from 34.7 mm preoperatively to 37.1 mm post-operatively, but decreased to 36.0 mm at the last follow-up (Table 2). The average loss of height of the fusion segment the PEEK/Ti/HA group and PEEK group was 1.2 mm and 1.1 mm, respectively (P > 0.05). Fortunately, both of the two patients with cage subsidence did not suffer any associated clinical symptoms and the intervertebral fusion of them was not interfered.

The restoration of physiological lordosis of the cervical spine is crucial to obtaining better dorsal shifting of the decompressed spinal cord and better postoperative clinical outcomes in ACDF [23, 24]. In this study, the segmental and overall cervical lordosis of all the patients were restored postoperatively and maintained well at the final follow-up (Table 2). These results were comparable with the previous studies [24, 25]. As for the clinical outcomes, the JOA and VAS scores were significantly improved after surgery in both the PEEK/Ti/HA and PEEK groups, and there was no significant difference between the two groups.

Our study had several limitations. First, the sample size was small and the follow-up time was short. Second, we did not enroll the patients with multi-level CDDD. So, future studies with larger numbers of patients with multi-level CDDD and longer follow-up period are needed.

## Conclusions

A PEEK cage with Ti and HA was successfully fabricated via a plasma spraying technique. In patients with single-level ACDF, PEEK cage coated with Ti and HA provided a higher fusion rate than uncoated PEEK cage at 3-month post-operation, while both of the two cages could achieve solid osseous fusion at the last follow up (100% fusion rate). Compared with the uncoated PEEK cage, PEEK/Ti/HA cage yielded similar favorable segmental and overall cervical lordosis, IH, and clinical outcomes after the surgery.

## Data Availability

Data will be available upon request to the corresponding author.

## Abbreviations

ACDF: Anterior cervical discectomy and fusion
CDDD: Cervical degenerative disc disease
Ti: Titanium
PEEK: Polyetheretherketone
CT: Computed tomography
HA: Hydroxyapatite
SEM: Scanning electron microscope
EDS: Energy disperse spectroscopy
XRD: X-ray diffraction
MTS: Mechanical testing machine
IH: Intervertebral height
C2-7a: C2-7 angle
SA: Segmental alignment
PACS: Picture archiving and communication systems
JOA: Japanese Orthopedic Association
VAS: Visual Analogue Scale
BMSCs: Bone mesenchymal stem cells
ALP: Alkaline phosphatase
BSP: Bone sialoprotein
